# Instagram Memes of Oral Nicotine Pouches: Content Analysis Study

**DOI:** 10.2196/84025

**Published:** 2026-03-24

**Authors:** Samia Amin, Yunping Wu, Samantha Baptista, Scott K Okamoto, Thaddeus A Herzog, Crissy Terawaki Kawamoto, Pallav Pokhrel

**Affiliations:** 1 Department of Biology, Southern Arkansas University, Magnolia, AR, United States; 2 Population Sciences in the Pacific Program, University of Hawaii Cancer Center, 701 Ilalo Street, Honolulu, HI, 96813-5516, United States, 1 8085645866

**Keywords:** Instagram, memes, nicotine, oral nicotine pouches, tobacco, Zyn

## Abstract

**Background:**

Oral nicotine pouches (ONPs), such as Zyn, have gained popularity among young people; however, their portrayal on social media remains under-studied. Instagram memes, a widely shared form of digital communication, may shape young people’s perceptions about ONPs and contribute to the widespread acceptance of ONP use.

**Objective:**

This study examines the thematic content of Instagram memes related to ONPs to understand how these products are represented online.

**Methods:**

The content of Instagram memes tagged with ONP-related hashtags—#oralnicotinepouch, #zyn, #on, #velo, and #nicotinepouch—was systematically analyzed. After screening, a total of 244 photo- and text-based memes were included in the final dataset. Using a structured coding framework, 3 researchers categorized the memes into key themes using NVivo software.

**Results:**

Three dominant themes emerged: (1) the Zyn community (35.6%)—memes fostered a sense of belonging among users; (2) marketing and branding (27.8%)—humorous critiques of product advertising and accessibility; and (3) perceived consequences of use (13.9%)—memes highlighted perceived positive or negative consequences of ONP use. Engagement metrics revealed high levels of interaction, with the Zyn community theme garnering the most user engagement.

**Conclusions:**

ONP-related Instagram memes are primarily focused on community identity, humor, and marketing, with community-centered content receiving the highest engagement. These findings indicate that social belonging and humor are central to the online representation of ONPs.

## Introduction

Oral nicotine pouches (ONPs) are a new type of tobacco product that, unlike traditional smokeless tobacco, use powdered nicotine rather than tobacco leaves [1]. They are typically used by placing them between the lip and gum, where they are held in the mouth to release nicotine [1]. The rising popularity of ONPs, particularly among young people, has drawn increasing attention from public health researchers and regulatory bodies [2]. Unlike traditional smokeless tobacco products, ONPs such as Zyn, On!, and Velo are marketed as tobacco-free alternatives that deliver nicotine without combustion, making them appealing to users seeking a discreet and convenient method of consumption [3]. However, short-term effects such as nausea, vomiting, bloating, and heartburn, along with long-term risks including nicotine addiction and potential oral health consequences, highlight the importance of understanding how these products are perceived in online environments [4,5]. These concerns are further amplified by the widespread perceptions of low harm of ONPs, influenced by their smokeless format and “tobacco-free” marketing [6]. Among various ONP brands, Zyn stands out as the most popular, widely recognized for its market dominance and consumer preference [7]. Despite the growing market presence of ONPs, at present, limited research has explored how ONPs are perceived and discussed within digital spaces, particularly through user-generated content on social media [7,8].

Most existing studies on ONPs have focused on marketing, branding, or sponsored content rather than user-generated discussions, leaving a gap in understanding how everyday users perceive and share these products online. On Reddit, prior research examined perceptions of ONPs through observational analysis of posts, revealing users’ experiences and attitudes toward these products [9]. On TikTok, studies have analyzed ONP-related videos, highlighting their growing popularity, the promotional strategies used, and patterns of user engagement [10-12]. Instagram (Meta Platforms, Inc), which has 3 billion monthly active users as of late 2025, serves as a key hub for the exchange of user-generated content, including nicotine consumption [13-16]. On Instagram, research has primarily focused on brand-marketed content, including visual advertisements and influencer posts illustrating how ONPs are promoted to large audiences [17]. Additional studies have examined ONP brand promotion through sponsorships and reward campaigns that encourage users to collect points for prizes; online retail tactics that leverage appealing product attributes such as flavors and brand reputation; and large-scale marketing campaigns across radio, television, and digital platforms, illustrating the diverse ways the ONP industry engages and interacts with consumers [18-20].

Research on Instagram posts more broadly shows how user-generated content can influence perceptions and social norms. Selfie-based portrayals on Instagram have demonstrated how smoking behaviors among young women can be glamorized through user-generated content, contributing to the normalization of tobacco use [15]. Similarly, Instagram posts related to e-cigarettes revealed that these products are framed as either harmful or socially acceptable [16]. Although prior research has examined ONP marketing content on Instagram [17], Instagram posts created by users, including memes, remain largely under-studied, representing an important gap in understanding peer-to-peer perceptions and social norms around ONP use.

Contents shared on Instagram as memes may especially influence perceptions and behavior related to nicotine products [21]. Memes are a widely shared and culturally relevant form of digital communication that usually uses wit and humor to further their content [22]. Young people often share tobacco- and marijuana-related memes with their friends, particularly memes that have received substantial attention in terms of likes and shares on social media [21]. However, how ONPs are depicted on Instagram memes remains unclear. As memes are an under-studied form of user-to-user communication, they can shape social norms, influence perceptions of product risk and acceptability, and provide early signals of emerging narratives that may inform prevention messages and policy-relevant communication strategies. Better understanding how the ONP-related content is represented in Instagram memes may provide valuable insights into the nature of the information about ONPs circulating on social media as well as potential social norms related to ONPs. To this end, this study seeks to conduct a systematic thematic content analysis of Instagram memes related to ONPs.

## Methods

### Ethical Considerations

This study was exempt from ethics review board approval because it involved the analysis of publicly available, anonymized data that did not include any identifiable private or sensitive personal information [23], consistent with previous tobacco-related social media research that has also been exempted from ethics approval for using public data [24,25].

### Meme Identification

A systematic search was conducted on Instagram in September 2024 using the hashtags #oralnicotinepouch, #zyn, #on, #velo, and #nicotinepouch. We selected Instagram because it is one of the most commonly used social media platforms by young people, and memes are a prominent and widely shared content type on Instagram [22,26]. All data included were publicly accessible through Instagram’s web interface. No private accounts or nonpublic posts were collected, ensuring compliance with Instagram’s user agreement. Data were extracted manually through systematic searches on the Instagram platform; no application programming interface was used. Each post was documented and stored in a secure database with screenshots and URLs to ensure reproducibility.

Approximately 1800 posts across 8 Instagram accounts associated with #oralnicotinepouch, #zyn, #on, #velo, and #nicotinepouch were screened by the second and third authors. The inclusion criteria required the memes to be photo based and text based and originate from specific Instagram accounts associated with ONP-related hashtags. Posts were reviewed independently by the 2 coauthors to identify the eligible memes. Duplicate posts (n=47), videos (n=1086), posts from deleted accounts (n=396), or posts from accounts without meme content (n=27) were excluded. A total of 244 photo- and text-based memes met the inclusion criteria and were incorporated into the final database for analysis. We selected both photo- and text-based memes for analysis because doing so would enable us to examine both visual and textual elements, which was expected to provide a comprehensive understanding of how the message was communicated in terms of language, tone, and impact.

### Codebook Development

The coding team consisted of 3 coauthors (YW, SB, and SA). Before coding commenced, the research team developed a detailed codebook. The team conducted a preparatory review of 10 randomly selected memes to familiarize themselves with the dataset and identify preliminary themes, visual styles, and recurring elements. The coding team collaborated using a structured approach to ensure consistency and accuracy in the analysis process.

For the development of the coding framework, the research team assigned each of the 3 coders (YW, SB, and SA) a subset of 30 randomly selected memes from the dataset. Each coder independently generated codes based on the primary themes and messages conveyed in the memes. Following this individual coding process, the team convened for a series of collaborative meetings to identify the most salient codes that aligned with the research objectives and grouped them into broader categories. Minor refinements were made to expand and remove a few codes based on team consensus, and all revisions were documented in an updated codebook to ensure transparency and auditability. The final codebook was organized into 3 major themes (Zyn community, marketing and branding, and perceived consequences of use), along with several additional but less prominent themes. A coding tree accompanied the codebook to ensure consistent application across the dataset. The codebook was developed primarily using an inductive approach; based on a careful review of a sample of memes, no deductive coding based on existing frameworks was applied [27,28]. The unit of analysis was each individual Instagram post containing memes. For carousel posts, the entire carousel was treated as 1 post, and only the first meme image was coded, as it is the primary visual content displayed to users. The complete codebook with operational definitions for each code is provided as Multimedia Appendix 1.

The team used qualitative analysis software (NVivo; Lumivero) to code the memes systematically, with 2 team members (YW and SB) independently coding each meme. Team meetings were conducted regularly, both virtually and in person, to discuss progress and refine the codebook. Shared resources, including the dataset and coding materials, were organized and stored on Google Drive for seamless access and collaboration. Discrepancies were addressed through group discussions. Once consensus was reached, the codes were finalized and stored in a centralized repository for review and further analysis. The final codebook was used to systematically code the 244 memes.

### Reliability and Validity

To ensure reliability, intercoder agreement using Cohen κ was periodically assessed throughout the coding process [29]. During pilot coding, Cohen κ ranged from 0.82 to 0.88 across all individual codes, and the mean κ of 0.85 (SD 0.02) was calculated for overall coding agreement, which demonstrated a high level of consistency and reproducibility in data categorization among all coders.

To maintain high construct validity, we ensured that the codes and categories accurately reflected the underlying concepts of health messaging, product promotion, and nicotine pouch use. This was done by obtaining continuous feedback from the research team and refining the codes based on the feedback [30].

### Engagement Metrics Analysis

Engagement metrics were extracted for the 3 most prominent themes (Zyn community, marketing and branding, and perceived consequences of use) by collecting the total number of likes, comments, and shares associated with posts in each theme. Cumulative engagement values were calculated to quantify overall user interaction across themes.

## Results

The majority of memes analyzed in this study were predominantly about Zyn, reflecting its strong presence on the Instagram platform. The analysis revealed a variety of themes with differing frequencies, reflecting the multifaceted nature of discussions surrounding ONP use.

### Engagement Metrics for the Top 3 Prominent Themes

The engagement metrics revealed that the top 3 themes driving interaction were Zyn community, marketing and branding, and perceived consequences of use. The Zyn community category garnered significant interaction, with 650,000 likes, 1600 comments, and 696,000 shares. Similarly, the marketing and branding category achieved 585,000 likes, 738 comments, and 497,000 shares. In comparison, the perceived consequences of use category recorded 215,000 likes, 224,000 comments, and 422 shares.

### Top 3 Prominent Themes

**Figure 1.**
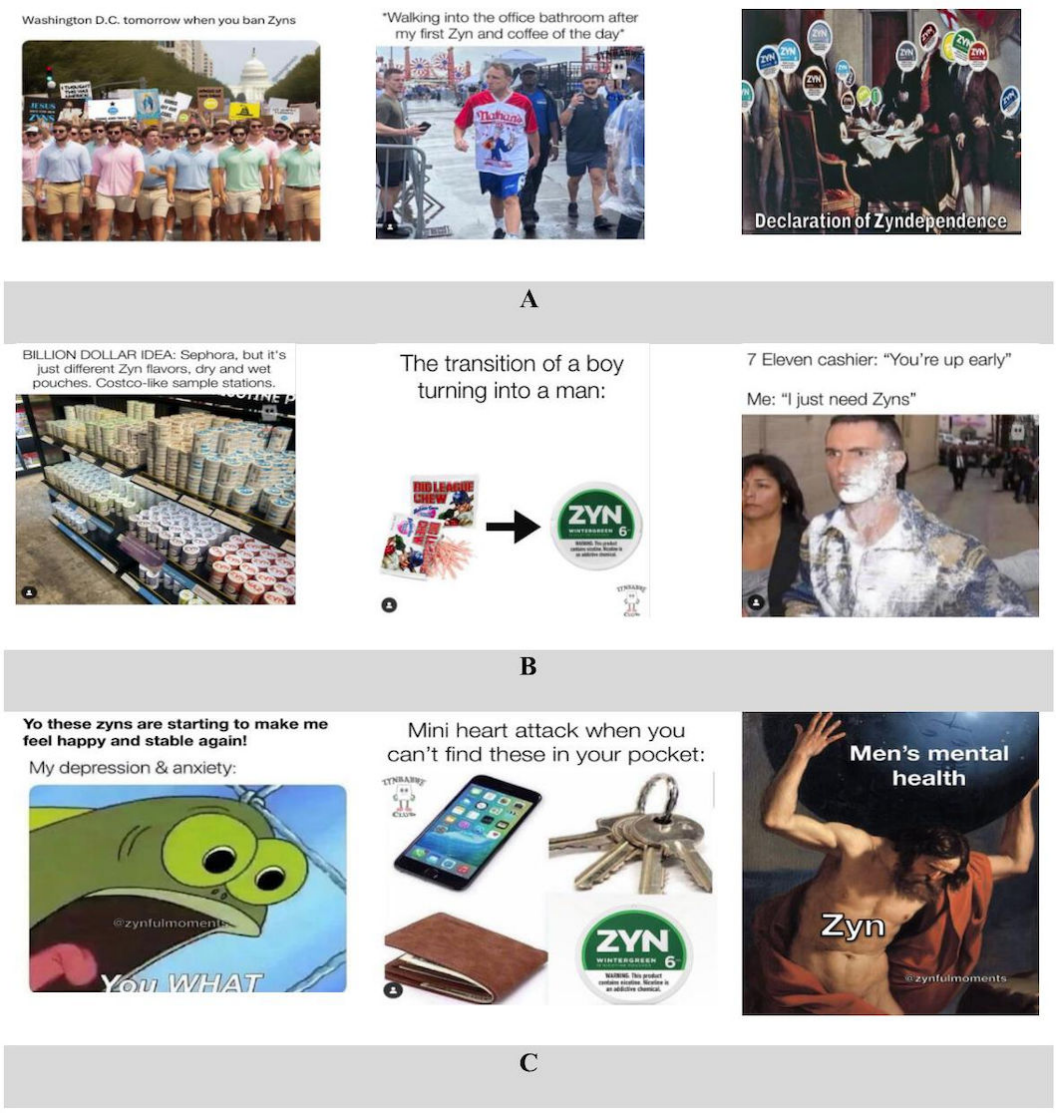
Memes that reflected these top 3 themes. (A) Sample memes illustrating the theme of Zyn community. (B) Sample memes illustrating the theme of marketing and branding. (C) Sample memes illustrating the theme of perceived consequences of use. Memes were posted by the following Instagram accounts, from left to right: (A), enhanced.snus [31], lippillowmaxxer [32], and the_zyn_head [33] (B), zyn_fluencers [34], zynbabweclub [35], zyncentral [36] (C), zynternet [37], enhanced.snus [31], zynfulmoments [38].

The most common meme category was Zyn community (87/244, 35.6%), followed by marketing and branding (68/244, 27.8%) and perceived consequences of use (34/244, 13.9%). Figure 1 shows a sample of memes that reflected these top 3 themes.

#### Theme 1: Zyn Community

The theme Zyn community highlighted the social aspect of Zyn use, where users developed group identity through their shared enthusiasm for the product. The memes in this category often combined humor with relatability to build a sense of belonging, allowing individuals to connect over their mutual use of Zyn. For example, a meme depicting people protesting in Washington, DC, after the Zyn ban tapped into the idea of collective defiance and solidarity within the Zyn user community. Another meme of a group of people staring at a man walking into the office bathroom after his first Zyn and coffee of the day highlights a sense of belonging among users who recognize and relate to the humorous scenario. Yet another meme showed a group of people facing Zyn and included the following text: “Declaration of Zyndependence.” This meme seemed to highlight a sense of unity and shared pride among Zyn users. In sum, these memes appeared to foster bonding among Zyn users by highlighting shared experiences and in-group jokes related to Zyn.

#### Theme 2: Marketing and Branding

The marketing and branding theme primarily focused on how Zyn and other nicotine pouch brands positioned themselves in the market. Memes often humorously highlighted the flashy advertising strategies, product packaging, and brand slogans used to appeal to consumers. For example, a meme stated, “Billion-dollar idea: Sephora, but it’s just a different Zyn flavor—dry and wet pouches. Costco sample station vibes,” thus humorously pointing out that Zyn’s marketing mimicked the aspirational branding strategies of high-end consumer goods. Another meme humorously showcased the transition from childhood to adulthood: “Big league chew to Zyn.” This meme reflects a generational shift from childhood to adulthood—referring to “Big League Chew,” a gum designed to mimic chewing Zyn, and portrays the transition to Zyn as a natural or expected part of growing up, potentially trivializing the shift to nicotine products. Other memes emphasized the ease of accessing Zyn and/or glorified the addiction to Zyn, for example, “7-Eleven cashier: You up early? Me: I just need Zyn.” Thus, these examples reflect how Zyn memes use humor to brand Zyn as an appealing product, glamorize Zyn use, and glorify the dependence on the product.

#### Theme 3: Perceived Consequences of Use

Several memes discussed the perceived consequences of using nicotine pouches, particularly in relation to mental health. One meme humorously stated, “Zyn are starting my day happy and stable again,” accompanied by an image of an animated character displaying a fearful, agitated expression, with the text “my depression and anxiety” displayed. Another meme humorously suggested that forgetting Zyn in one’s pocket—just like a phone or wallet—could cause a “mini heart attack,” emphasizing the intensity of dependence on the Zyn product. This exaggeration highlights how users may jokingly acknowledge their reliance on ONP products while continuing to engage with them.

Finally, a meme portrayed a man labeled as “Zyn” lifting a large boulder representing stress or negative affect. This meme seemed to communicate that the use of Zyn helped cope with high levels of stress or negative affect. Alternatively, the boulder may be interpreted to indicate high levels of dependence on Zyn. In sum, these memes reflect a mix of perceived positive and negative consequences associated with ONP use.

### Additional Themes

**Figure 2.**
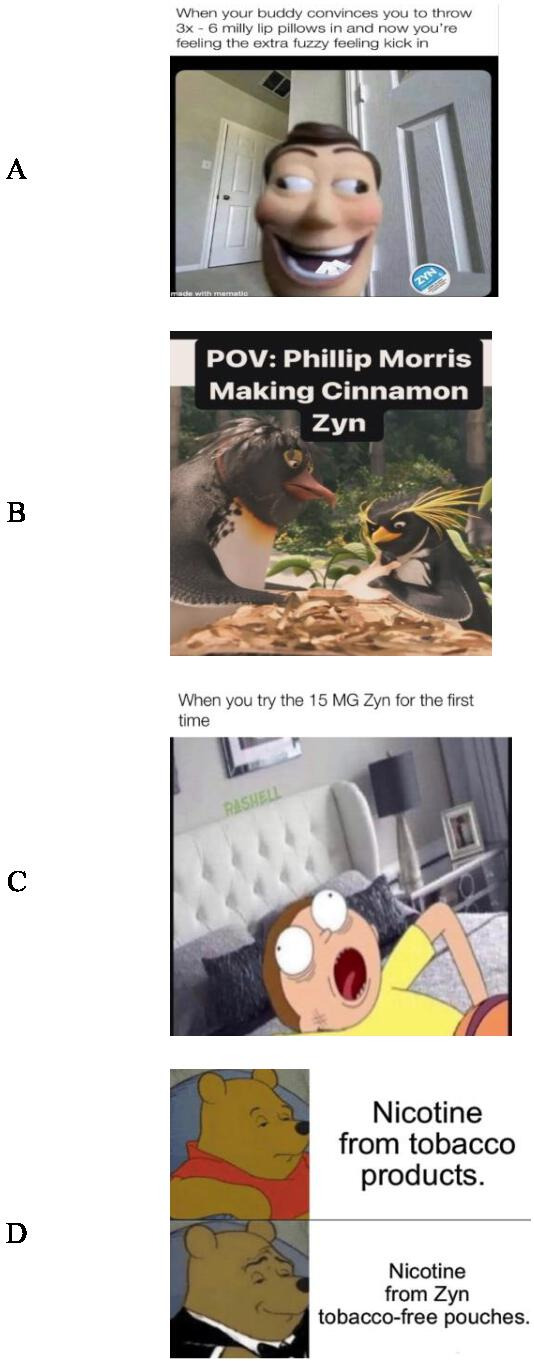
Memes that reflected additional themes. (A) Memes related to nicotine strength. (B) Memes related to flavor varieties. (C) Memes related to first-time user experiences. (D) Memes related to comparisons between Zyn and other tobacco products. Memes were posted by the following Instagram accounts: (A) lippillowmaxxer [32], (B) the_zyn_head [33], (C) zyncentral [36], and (D) zynternet [37].

Other themes identified in the analysis included nicotine strength, flavor varieties, first-time user experiences, and comparisons between Zyn and other tobacco product. When combined, these themes represent a minor portion of the overall dataset, contributing approximately 22.5% (55/244) of the total samples. Figure 2 shows a sample of memes that reflected these additional themes. Engagement metrics for other themes were not reported due to lower levels of interaction.

## Discussion

### Principal Findings

This study explored ONP-related memes on the social media platform Instagram. The analysis revealed 3 prominent themes across the memes: Zyn community, marketing and branding, and perceived consequences of use. Memes in the Zyn community theme highlighted social bonding and shared experiences among users, while marketing and branding evaluated the potential appeal of ONPs. The perceived consequences of use theme captured the mixed perceptions of potential consequences of ONP use, such as stress or affect regulation and dependence. Additional themes, including nicotine strength, flavor varieties, first-time user experiences, and comparisons between Zyn and other tobacco products, were less prevalent.

Emerging tobacco products such as e-cigarettes are increasingly shaped by digital culture, where social media communities play a critical role in influencing how these products are perceived, shared, and normalized. Prior research on vaping and ONP-related social media content closely aligns with the Zyn community theme in this study, demonstrating how humor, shared narratives, and visually engaging posts foster a sense of belonging and normalize product use. Humor-driven content has been shown to strengthen community connections [39], while in-group communication and youth-friendly imagery help promote shared identity and normalize vaping behaviors [40]. Similarly, visually appealing posts such as selfies and product photos reinforce community bonds [41]. This pattern extends to ONPs, with Reddit discussions framing ONPs as lifestyle enhancers and TikTok content portraying them as trendy and youth-oriented through humorous, relatable narratives [9-11]. Our findings show that Instagram memes replicate these mechanisms, positioning ONPs as products that facilitate social connection and identity formation.

Advertisement, brand promotion, and marketing of e-cigarettes on social media have become prevalent practices [42-44]. Emerging evidence shows that ONPs are promoted on social media in ways similar to other tobacco products, such as e-cigarettes, with content framing them as socially acceptable, lifestyle-oriented, and often humorous, appealing to younger audiences [9-11]. Studies have shown that images depicting vaping activities tend to generate high engagement and that platforms such as Instagram and Pinterest are effective channels for e-cigarette product displays [11,42]. These findings align with our study’s marketing and branding theme, which highlights Zyn’s marketing strategies, particularly through memes that humorously position the brand as aspirational and ubiquitous, while also suggesting that Zyn use is a grown-up behavior. Research studies also indicate that ONPs are marketed for noncessation purposes and commonly use youth-targeted cues, lifestyle imagery, and implicit health messaging [12,17]. Collectively, these findings reinforce that ONP marketing on social media, much like e-cigarette marketing, leverages visual storytelling, humor, and lifestyle branding to broaden appeal, particularly among younger and digitally engaged audiences.

The perceived consequences of use theme reflects the mixed beliefs surrounding ONP use. Consistent with recent TikTok and Reddit studies, our findings show that users often frame ONPs as tools for stress relief or affect regulation, despite contradictory evidence [9,10]. Memes in this category frequently portray Zyn as a coping mechanism for stress, affect, or poor mental health symptoms (eg, anxiety and depression) [11] or a humorous dependency (eg, the “mini heart attack” when a pouch is missing). These portrayals align with findings from nicotine research indicating that users often perceive nicotine as stress reducing, although nicotine can exacerbate stress and withdrawal symptoms [45]. The humorous framing in memes may obscure these harms, reinforcing the normalization and trivialization of dependence.

The findings from this study highlight significant implications for public health strategies targeting ONP use. First, given the widespread use of humor and relatability in memes, public health campaigns could consider adopting similar strategies to effectively communicate the health risks associated with ONPs. By leveraging humor, these campaigns may better engage younger and more digitally connected audiences, potentially reshaping the cultural narratives surrounding nicotine use. Second, the study emphasizes the need for policies that address the normalization of these products in online spaces. Regulatory agencies should recognize memes and other user-generated content as influential promotional tools and consider monitoring these platforms, as understanding how memes reinforce marketing tactics and community identities can inform regulatory frameworks aimed at controlling the promotion and accessibility of these products, particularly on youth-oriented digital platforms. At the same time, public health professionals have an opportunity to engage with active communities, such as the online Zyn community on Instagram, using their language and humor to provide accurate, balanced, and reliable information about nicotine, including both addiction risks and potential harm-reduction aspects of noncombustible products. Third, policies should also target indirect marketing practices, including influencer partnerships, trend-based content, and meme-driven brand symbolism, which often escape traditional advertising oversight. By combining engaging public health messaging with updated regulatory measures, it may be possible to reduce the appeal of ONPs among adolescents and young adults.

### Strengths, Limitations, and Future Directions

This study has several strengths that enhance its relevance to tobacco research. First, it investigates the growing online community of ONP users, using memes that capture the social dynamics and cultural narratives surrounding these products. The analysis also examines how memes blend humor and social commentary, offering insights into user perceptions and behaviors. This is particularly relevant to young people—a group especially vulnerable to nicotine initiation and addiction—as such content may reinforce interest in and normalize nicotine pouch use. By focusing on the popular social media platform Instagram, this study highlights the need for innovative public health interventions that resonate with digital communities. Furthermore, the research sheds light on the influence of user-generated content in shaping product perceptions and cultural identities, contributing to the broader conversation about how digital platforms impact health behaviors.

While our study offers valuable insights into the social and cultural dimensions of ONP use, there are several limitations to consider. The analysis was limited to Instagram, which was selected due to its popularity among young adults and its prominence in visual meme-based content. However, nicotine-related messaging also appears on platforms such as TikTok, X (formerly Twitter), and Reddit, which were not examined here. The reliance on memes as a primary data source may also have introduced skewed perceptions, as memes often exaggerate or trivialize experiences for comedic effect and may not fully capture the complexities of user experiences or broader attitudes. The hashtag-based sampling strategy may have excluded relevant content outside those parameters and increased the likelihood of including posts from nonhuman accounts, such as automated promotional profiles. Additionally, posts were manually screened, and lower-engagement content was removed, which limits reproducibility and generalizability and may have omitted important perspectives. Finally, the engagement matrix was reported as cumulative totals per theme, which may limit variation at the individual post level.

Future research should explore cross-platform differences in nicotine-related discourse to develop a more comprehensive understanding of how ONPs are represented and circulated online. Combining meme analysis with traditional qualitative methods, such as focus groups or in-depth interviews with members of digital communities, could provide a more nuanced understanding of how ONP use is perceived across different demographic groups. Incorporating bot detection tools and broader sampling strategies could help improve the rigor of future analyses. Studies using post-level engagement data and applying descriptive or inferential statistical techniques may more accurately characterize patterns of user interaction. Additionally, longitudinal designs could help track evolving cultural perceptions of nicotine products as they become more mainstream, and future work may also examine how memes contribute to the adoption of other emerging nicotine products, such as smokeless and heated tobacco products.

### Conclusions

Our study illustrates how memes reflect the perceptions of ONPs, emphasizing key themes of community, marketing, and consequences of use. The findings highlight the importance of understanding the digital culture surrounding these products, particularly as they gain popularity among younger, more digitally connected populations. By recognizing the role of humor, relatability, and branding in influencing attitudes, public health campaigns can be better tailored to address the cultural appeal of ONPs and mitigate their potential harm and understand their potential as substitutes for cigarettes. The study lays the groundwork for future research that can explore the intersection of digital culture, substance use, and public health, offering valuable insights for designing more effective, culturally relevant health interventions and providing reliable, balanced, and evidence-based information on nicotine and tobacco products.

## Supplementary material

10.2196/84025 Multimedia Appendix 1 Comprehensive hierarchical codebook detailing themes, operational definitions, indicators, and example applications used to systematically guide and standardize the qualitative analysis.

## Abbreviations

ONP: oral nicotine pouch
