# Supplementary material for: Instagram Memes of Oral Nicotine Pouches: Content Analysis Study
Source: Online J Public Health Inform. 2026 Mar 24;18:e84025. doi: 10.2196/84025 (PMC13012227; doi:10.2196/84025)
Supplement: Multimedia Appendix 1 [file ojphi-v18-e84025-s001.pdf]

Supplementary Appendix1: Codebook and operational definitions

Overview: This appendix provides the complete codebook used in the analysis, including operational definitions and example indicators for each code. Codes are organized under the three major themes (Zyn community, Marketing and branding, Perceived consequences of use) and four additional minor themes (Nicotine strength, Flavor varieties, First-time user experiences, Comparisons with other tobacco products).

Coding structure: A hierarchical coding tree was developed to support consistent application across the dataset. All codes, definitions, and applied examples were reviewed iteratively and refined through team consensus.

1. **Theme 1**: Zyn community: Codes capturing social identity, group belonging, and shared experiences among Zyn users.
    i. Group identity definition: Memes that portray Zyn users as part of a collective or in-group. Indicators: References to "Zyn community," unity, pride, or belonging.
    ii. Shared experiences definition: Humor or scenarios highlighting relatable daily experiences using Zyn. Indicators: First Zyn of the day, workplace or school situations.
    iii. Solidarity and collective action definition: Depictions of users supporting or defending the product. Indicators: Protests, collective defiance, group camaraderie.
    iv. In-group humor definition: Jokes understood primarily by Zyn users. Indicators: "Zyndependence," ongoing inside jokes.
2. **Theme 2**: Marketing and branding: Codes reflecting how Zyn and other ONP brands are positioned, promoted, or perceived.
    i. Product promotion definition: Content highlighting branding messages or advertising styles. Indicators: Memes referencing slogans, flashy advertising.
    ii. Packaging and product appeal definition: Emphasis on design, packaging, or product presentation. Indicators: Aesthetic comparisons, beauty-industry humor.
    iii. Accessibility and convenience definition: Portrayals of ease of obtaining or using Zyn. Indicators: Retail jokes (e.g., "I just need Zyn" at 7-Eleven).
    iv. Normalization or glamorization definition: Content making Zyn use appear desirable, trendy, or aspirational. Indicators: Comparisons to luxury goods, lifestyle integration.
3. **Theme 3**: Perceived consequences of use: Codes related to mental, emotional, or physical experiences tied to Zyn use.
    i. Stress relief definition: Memes suggesting Zyn helps manage stress or negative emotions. Indicators: Visual metaphors like lifting boulders labeled stress.
    ii. Mental health jokes definition: Humor about emotional regulation or mood improvement. Indicators: Anxiety, depression, stability references.
    iii. Dependence or overreliance definition: Exaggerated reactions to not having Zyn available. Indicators: "Mini heart attack" when forgetting a pouch.
    iv. Dual interpretations (positive and negative) definition: Memes acknowledging both benefits and potential harms. Indicators: Mixed messaging on coping and dependence.

Additional minor themes

i. Nicotine strength definition: Mentions of different strength levels or reactions to strong pouches. Indicators: Shock, excitement, or humor about high nicotine doses.

ii. Flavor varieties definition: References to preferences or comparisons between flavors. Indicators: Jokes about choosing flavors, flavor-based identities.

iii. First-time user experiences definition: Depictions of initial reactions to trying Zyn. Indicators: Unexpected intensity or humorous struggles.

iv. Comparisons with other tobacco products definition: Contrasts between Zyn and cigarettes, vapes, or other nicotine products. Indicators: Portrayals of switching, evolution, or replacement.
